# LncRNA GAS5 promotes apoptosis as a competing endogenous RNA for miR-21 via thrombospondin 1 in ischemic AKI

**DOI:** 10.1038/s41420-020-0253-8

**Published:** 2020-04-02

**Authors:** Xuemei Geng, Nana Song, Shuan Zhao, Jiarui Xu, Yong Liu, Yi Fang, Mingyu Liang, Xialian Xu, Xiaoqiang Ding

**Affiliations:** 1 Department of Nephrology, Zhongshan Hospital, Fudan University; Shanghai Institute of Kidney and Dialysis; Shanghai Key Laboratory of Kidney and Blood Purification; Shanghai Medical Center of Kidney Disease, Shanghai, China; 2 Department of Physiology and Center of Systems Molecular Medicine, Medical College of Wisconsin, Milwaukee, WI USA

**Keywords:** Acute kidney injury, Long non-coding RNAs, Gene regulation

## Abstract

Mounting evidence has indicated that long noncoding RNAs (lncRNAs) and microRNAs (miRNAs) played important roles in renal ischemia/reperfusion (I/R) injury. However, the involvement of lncRNA growth arrest specific 5 (GAS5) in acute kidney injury (AKI) remained largely unexplored. This study aimed to determine possible mechanisms of GAS5 in the renal I/R process. We found that GAS5, noticeably upregulated by renal I/R injury, was further suppressed by delayed IPC while knockdown of miR-21 in vivo before IPC could significantly increased the GAS5 levels. Concurrently, TSP-1 was negatively regulated by miR-21 in vivo and vitro. Additionally, Reciprocal repression of GAS5 and miR-21 was identified. Knockdown of miR-21 in H6R0.5 treated HK-2 cells promoted apoptosis. Co-transfection of miR-21 mimic and pcDNA-GAS5 or pcDNA-Vector were performed, results of which showed that inhibition of miR-21 on TSP-1 could be rescued by overexpression of GAS5. This study suggested that GAS5 facilitated apoptosis by competitively sponging miR-21, which negatively regulated TSP-1 in renal I/R injury. This novel regulatory axis could act as a therapeutic target for AKI in the future.

## Introduction

Acute kidney injury (AKI) is a common complication characterized by a decline in renal function. Renal ischemia/reperfusion (I/R) insult contributes greatly to AKI. However, the established underlying mechanisms of I/R injury are only the tip of the iceberg. Cell apoptosis is considered to be involved in pathogenesis of I/R injury. It has been reported that many mechanisms contribute to apoptosis regulation in renal tubular epithelial cells in the process of renal I/R^1^.

miR-21, an important hypoxia-responsive miRNAs, was originally found in a cancer study due to its anti-apoptotic function. Our work demonstrated that miR-21 contributed to renal protection by reducing apoptosis via inhibiting programmed cell death protein 4 (PDCD4)^2^ and phosphatase and tensin homolog deleted on chromosome ten (PTEN)^3^. Thrombospondin-1 (TSP-1) with anti-angiogenesis effect, as a novel target gene of miR-21 in vascular endothelial cells^4^, also has pro-apoptosis function reported in the renal tubular epithelial cells from the study by Thakar et al.^5^.

Long noncoding RNAs (lncRNAs) are a novel class of non-protein-coding RNAs whose transcripts are over 200 nt in length. Emerging evidence has shown that lncRNAs are involved in many biological processes by regulating gene expression at epigenetic, transcriptional, and post-transcriptional levels^6,7^. Hypoxia-regulated lncRNAs were identified in some tumor studies^8^ and an effect of I/R on lncRNAs expression was uncovered as well^9^. Ectopic expression of lncRNA GAS5 was first found in hypoxia-treated renal tubular epithelial cells in Yu’s study^10^. Our previous study suggested that GAS5 might contribute to apoptosis in renal I/R injury^11^. However, how GAS5 regulates apoptosis induced by renal I/R remains unclear. In addition, the negative regulation of GAS5 by miR-21 was found in breast cancer^12^. Thus, in this study, we will explicit: (1) the protection of miR-21 in renal I/R injury-induced cell apoptosis might be related with inhibition of GAS5 and TSP-1, (2) GAS5 as a competing endogenous RNA (ceRNA) of miR-21 might contribute to renal I/R injury by regulating expression of TSP-1.

## Results

### miR-21, GAS5, and TSP-1 expression and apoptosis in mice kidneys after I/R

**Fig. 1:**
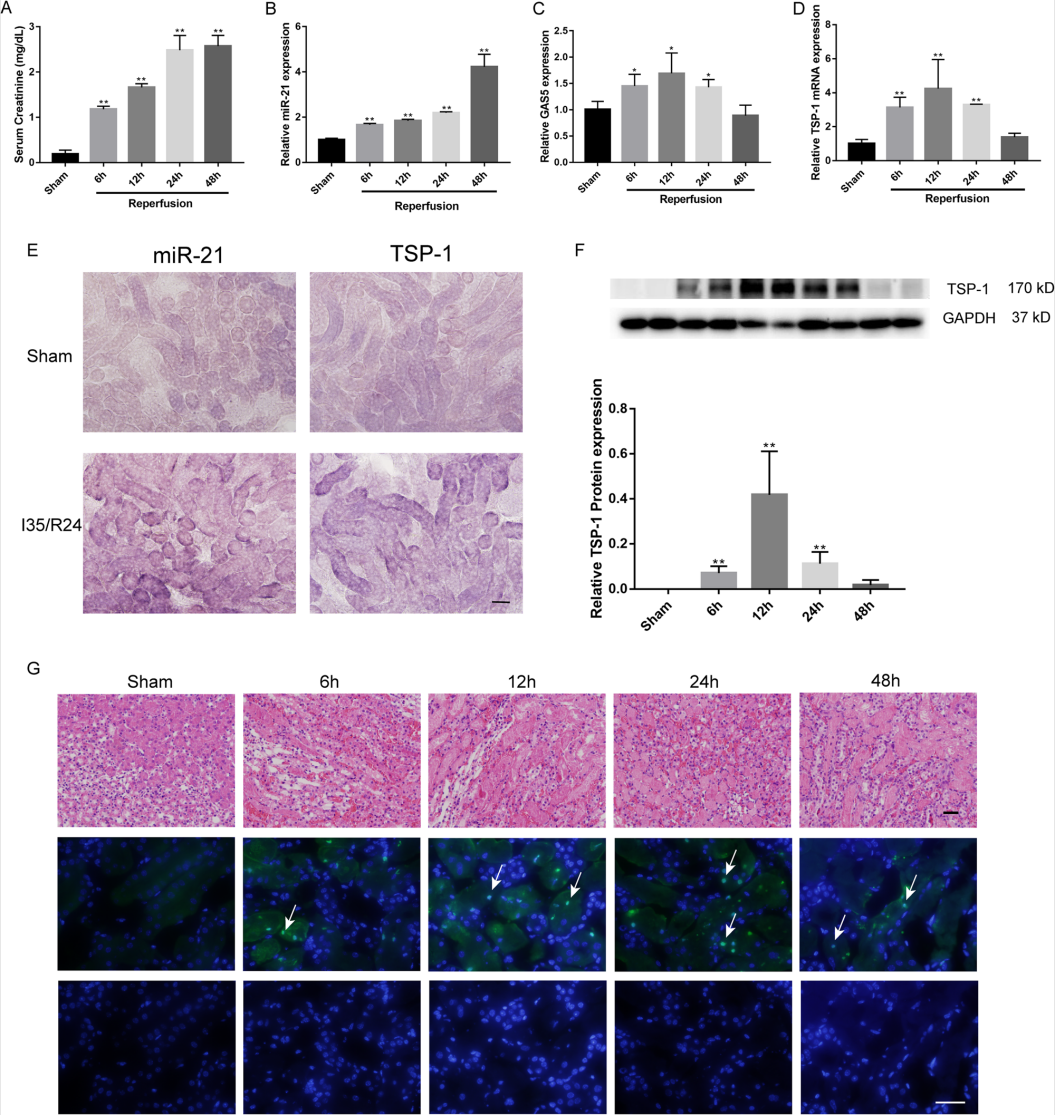
Acute kidney injury induced by renal ischemia and reperfusion. The levels of serum creatinine (**a**), miR-21 (**b**), GAS5 (**c**), TSP-1 mRNA (**d**), and TSP-1 protein (**f**) in mice subjected to renal ischemia for 35 min, followed by various reperfusion time. **e** Representative image of ISH for miR-21 and TSP-1 (photographed at ×20 magnification). **g** Histopathological changes (×20 magnification) and apoptosis in mice kidneys induced by renal ischemia and reperfusion (×40 magnification) evaluated by the TUNEL method. *N* = 4, ^*^P < 0.05, ^**^P < 0.01 vs. the Sham group. Scale bars, 100 μm.

In our previous study, we found that lncRNA GAS5 might contribute to renal I/R injury due to its pro-apoptosis function^11^. However, the mechanism of GAS5 involvement in I/R-induced apoptosis remains unknown. The expression levels of miR-21, GAS5, and TSP-1 in mice kidneys were detected at diverse reperfusion intervals (6, 12, 24, and 48 h) after ischemia for 35 min. As illustrated in Fig. 1a, the levels of serum creatinine were significantly higher in renal I/R groups than that in Sham group.

The time-course analysis indicated that mRNA levels of GAS5 and TSP-1 were increased after I/R and peaked at 12 h of reperfusion, and miR-21 expression increased gradually until 48 h of reperfusion (Fig. 1b–d). The protein expression of TSP-1 was almost undetectable in Sham-operated kidneys, peaked at 12 h and decreased to the baseline level at 48 h of reperfusion, which was consistent with our RT-PCR results and similar to the results from Thakar’s study (Fig. 1f). miR-21 and TSP-1 in I/R (24 h) treated kidneys were expressed mainly in renal tubular epithelial cells, as shown in the ISH results (Fig. 1e). Therefore, we hypothesized that a relationship among miR-21, GAS5, and TSP-1 might exist.

In addition, our results revealed that the percentages of apoptotic cells in mice kidney tissues were higher in the I/R groups than that in the Sham group, as well as the severity of renal injury assessed by histopathological manifestations (Fig. 1g).

### The preconditioning-induced upregulation of miR-21 accompanied by downregulated expression of GAS5 and TSP-1 contributed to renal protection of delayed IPC

**Fig. 2:**
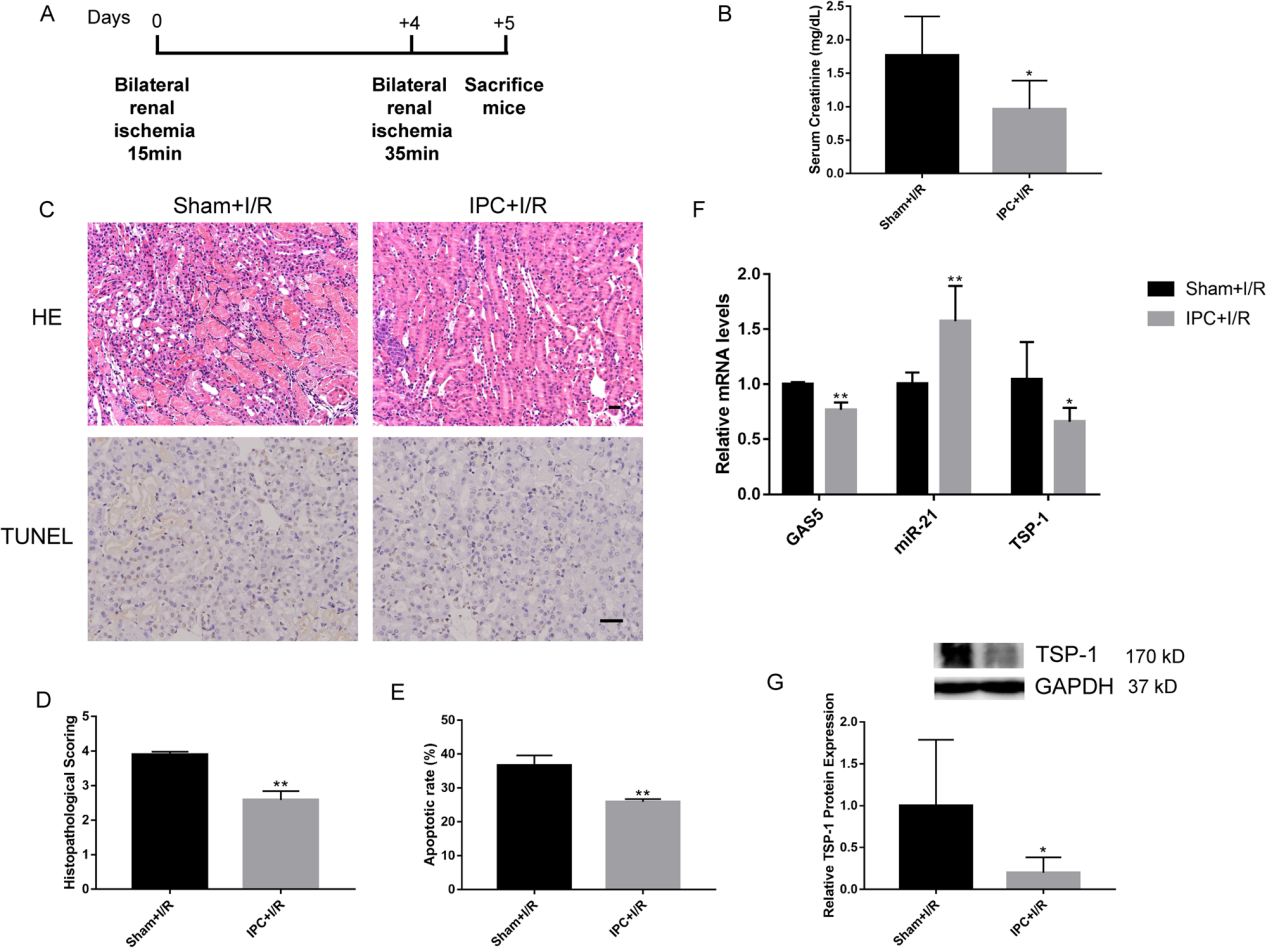
Delayed IPC alleviated ischemic AKI. **a** Schematic diagram depicting the animal treatment procedure. **b** Measurement of serum creatinine levels showed that ischemic AKI was alleviated by delayed IPC (*N* = 5–6). **c** Representative images of renal H&E (×10 magnification) and TUNEL staining (×20 magnification). Histopathological scores (**d**) (*N* = 5–6) and apoptotic rates (**e**) (*N* = 3) were lower in the IPC+I/R group than those in the Sham+I/R group. **f**, **g** Delayed IPC upregulated miR-21 and downregulated GAS5 and TSP-1 levels induced by renal I/R injury (*N* = 5–6). ^*^P < 0.05, ^**^P < 0.01 vs. the Sham+I/R group. Scale bars, 100 μm.

To further determine the pro-apoptotic function of GAS5 in ischemic AKI and the relationship among the three genes, mice were divided into two groups: an IPC+I/R group and a Sham+I/R group. A schematic diagram depicting the animal treatment procedure was demonstrated in Fig. 2a. Similar to our previous studies^2^, IPC significantly improved renal function and markedly alleviated histopathologic damage (Fig. 2b–d), as well as decreased the percentages of apoptotic tubular cells (Fig. 2e). Additionally, miR-21 levels in mice kidneys were remarkably higher for the IPC + I/R group than the Sham+I/R group, accompanied by downregulated TSP-1 mRNA and protein expressions (Fig. 2f, g). GAS5 expression induced by I/R insult in mice renal tissues was significantly downregulated by delayed IPC (Fig. 2f), which suggested a possible negative correlation between GAS5 and miR-21.

### Knockdown of miR-21 promoted apoptosis might by upregulating GAS5 and TSP-1 expressions in the delayed IPC

**Fig. 3:**
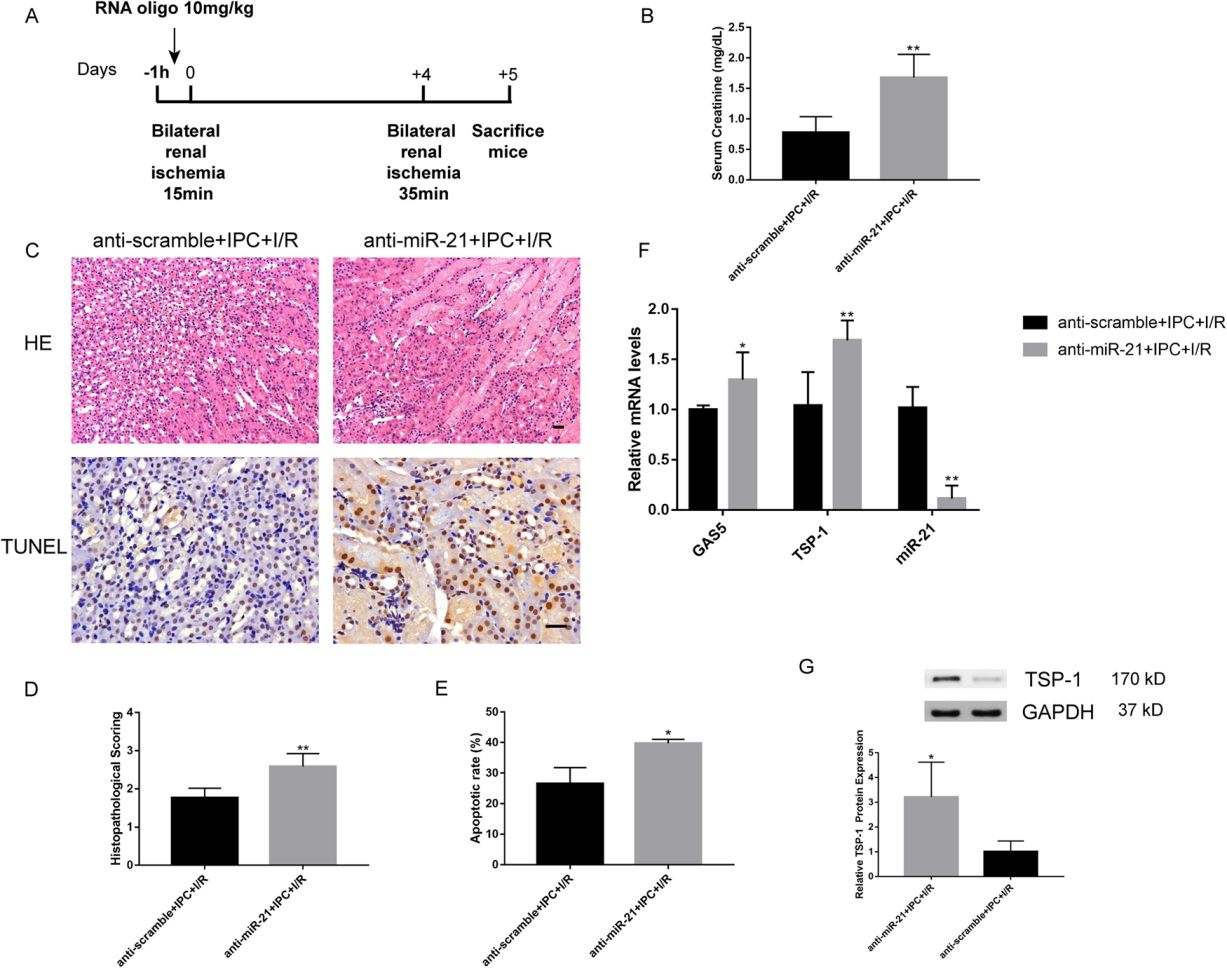
Inhibition of miR-21 exacerbated ischemic AKI. **a** Schematic diagram depicting the animal treatment procedure. **b** Knockdown of miR-21 in mice exposed to delayed IPC remarkably increased serum creatinine levels (*N* = 6). **c** Representative images of renal H&E (×10 magnification) and TUNEL staining (×20 magnification). Histopathological scores (**d**) (*N* = 6) and apoptotic rates (**e**) (*N* = 3) were higher in the anti-miR-21+IPC+I/R group than those in the anti-scramble+IPC+I/R group. **f** Inhibition of miR-21 significantly increased GAS5 and TSP-1 mRNA levels (*N* = 6). **g** Knockdown of miR-21 upregulated TSP-1 protein expression (*N* = 4). ^*^P < 0.05, ^**^P < 0.01 vs. anti-scramble+IPC+I/R group. Scale bars, 100 μm.

miR-21 knockdown has been suggested to attenuate the renal protection conferred by IPC. As shown in Fig. 3b–e, the serum creatinine level, renal histological injury score and percentages of apoptotic cells at 24 h of the second reperfusion in mice receiving LNA anti-miR-21 were significantly higher than those in mice with anti-scramble treatment. Concurrently, effective inhibition of miR-21 increased the mRNA and protein expressions of targeted TSP-1 (Fig. 3f, g). In addition, Fig. 3f showed that GAS5 levels were significantly higher in the anti-miR-21+IPC+IR group than in the control group. The results suggested that the protective effect of miR-21 might be mediated not only by targeted TSP-1 but also by GAS5 attenuating tubular cell apoptosis.

### TSP-1, a target of miR-21, contributed to H/R-induced apoptosis in renal proximal tubular epithelial cells

**Fig. 4:**
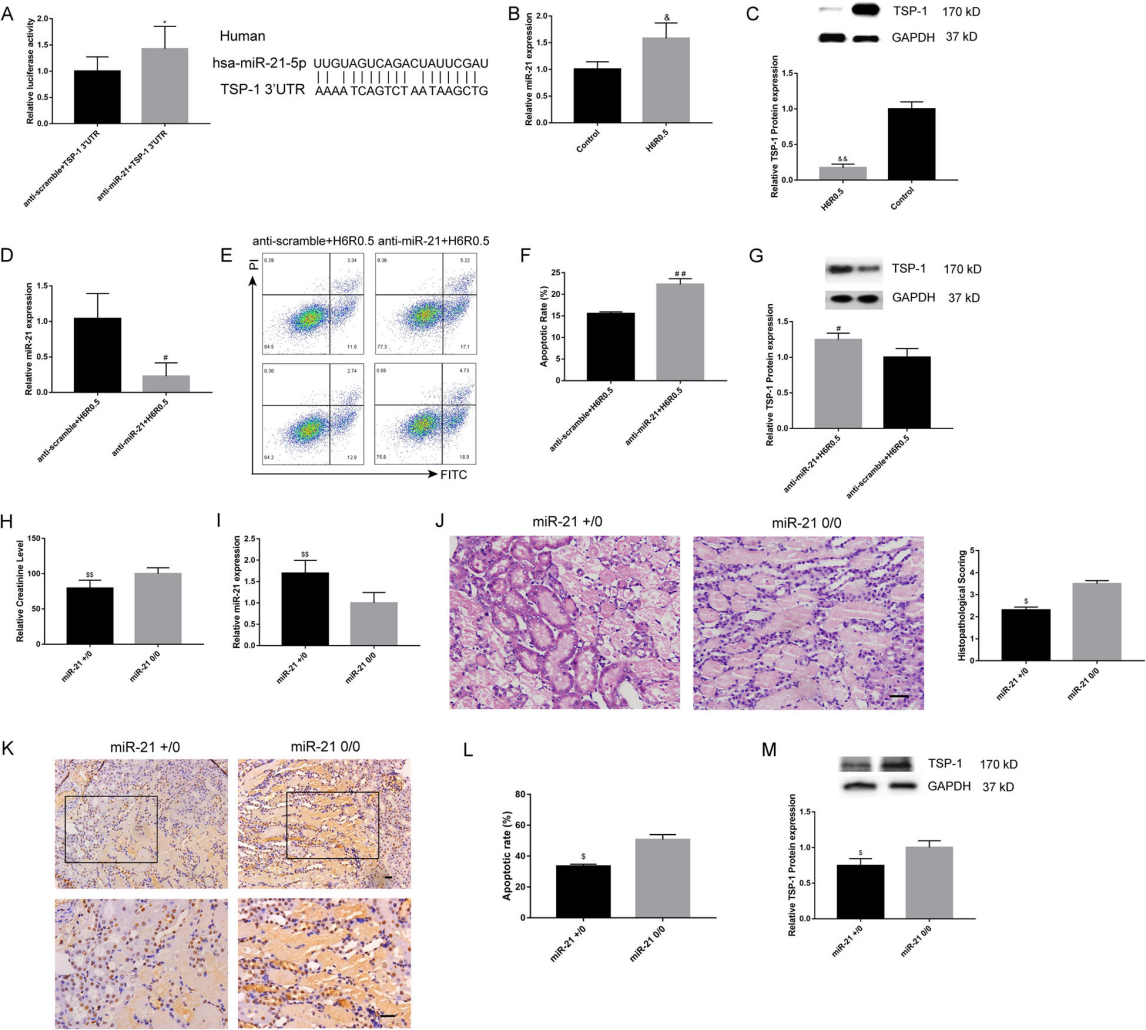
miR-21 targeted TSP-1 might contribute to H/R or renal I/R treatment-induced apoptosis. **a** Luciferase reporter assays confirmed an interaction between miR-21 and the 3′-UTR of TSP-1 mRNA. *N* = 7, *P < 0.05 vs. anti-scramble+TSP-1 3′UTR. miR-21 was upregulated (**b**), and the TSP-1 protein was downregulated (**c**) by H6R0.5 treatment in HK-2 cells, *N* = 3, ^&^*P* < 0.05, ^&&^*P* < 0.01 vs. the Control. Effective knockdown of miR-21 by LNA anti-miR-21 (**d**) exacerbated H6R0.5-induced apoptosis (**e**, **f**) and increased the protein expression of TSP-1 (**g**), *N* = 3, ^#^*P* < 0.05, ^##^*P* < 0.01 vs. anti-scramble+H6R0.5. **h**, **i** Serum creatinine levels were lower in miR-21+/0 mice with higher miR-21 levels (*N* ≥ 4). **j**–**m** miR-21+/0 mice presented lower histopathological scores, lower apoptotic rates and downregulated TSP-1 protein expression compared with miR-21 0/0 mice (*N* = 2–4), ^$^*P* < 0.05, ^$$^*P* < 0.01 vs. the miR-21 0/0 group. Scale bars, 100 μm.

Bioinformatic analysis was employed to predict the interaction between miR-21 and the 3′-UTR of TSP-1 (Fig. 4a). As demonstrated by luciferase reporter assays, co-transfection of anti-miR-21 and the TSP-1 3′UTR reporter construct group had markedly higher luciferase activity compared with co-transfection of anti-scramble group, which revealed that miR-21 might regulate the expression of TSP-1 by interactions with the 3′UTR.

**Fig. 5:**
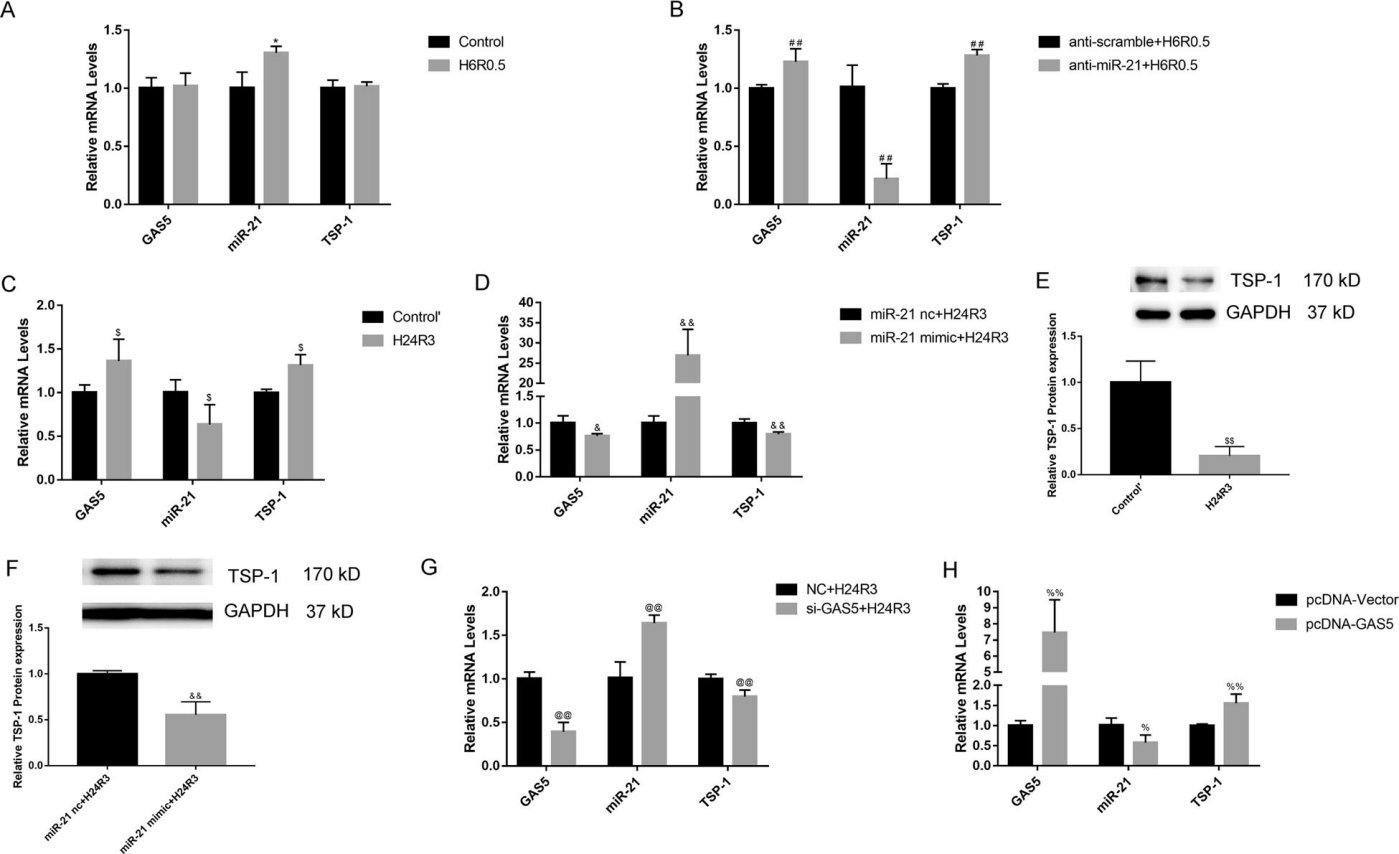
Mutual regulation among GAS5, miR-21 and TSP-1. **a** H6R0.5 treatment induced an increase in miR-21 but did not change the levels of GAS5 and TSP-1. **b** Knockdown of miR-21 by LNA anti-miR-21 noticeably upregulated GAS5 and TSP-1 expression under H6R0.5 condition. **c** H24R3 treatment downregulated miR-21 expression and upregulated GAS5 and TSP-1 mRNA expressions. **d** Overexpression of miR-21 under H24R3 condition decreased GAS5 and TSP-1 mRNA expressions. **e** TSP-1 protein levels were downregulated by H24R3. **f** Overexpression of miR-21 decreased the TSP-1 protein levels under H24R3 condition. **g** Knockdown of GAS5 upregulated miR-21 expression and downregulated TSP-1 expression under H24R3 condition. **h** Overexpression of GAS5 downregulated miR-21 expression and increased TSP-1 levels. *N* = 3–4, ^*^P < 0.05 vs. Control, ^##^*P* < 0.01 vs. anti-scramble +H6R0.5, ^$^*P* < 0.05, ^$$^*P* < 0.01 vs. Control′, ^&^*P* < 0.05^, &&^*P* < 0.01 vs. miR-21 nc+H24R3, ^@@^*P* < 0.01 vs. NC + H24R3, ^%^*P* < 0.05, ^%%^*P* < 0.01 vs. pcDNA-Vector.

Additionally, H6R0.5 insult significantly upregulated the expression of miR-21 and downregulated TSP-1 protein (Fig. 4b, c) but not TSP-1 mRNA levels in HK-2 cells (Fig. 5a). Furthermore, the effective knockdown of miR-21 in H6R0.5-treated HK-2 cells by the LNA anti-miR-21 increased the expression of TSP-1 protein and apoptotic cell percentages (Fig. 4d–g). Thus, these data suggested that knockdown of miR-21 could promote apoptosis in HK-2 cells via its target TSP-1.

We further verified the regulation of TSP-1 by miR-21 in miR-21+/0 mice. As illustrated, renal injury induced by I/R was ameliorated in miR-21+/0 mice compared with miR-21 0/0 mice, indicated by serum creatinine levels, histopathological scoring and TUNEL apoptotic cell percentages (Fig. 4h–l). Concurrently, TSP-1 protein expression was downregulated upon miR-21 upregulation in miR-21+/0 mice kidneys (Fig. 4m). Thus, in vivo and in vitro, we reconfirmed the negative regulation of TSP-1 by miR-21 in renal I/R injury.

### Effects of reciprocal repression between miR-21 and GAS5 on apoptosis and TSP-1 expression in vitro

GAS5 levels were upregulated in the H6R0.5-treated cells with LNA anti-miR-21 (Fig. 5b), although H6R0.5 insult did not alter the expression of GAS5 (Fig. 5a). H24R3 was also established due to decreased miR-21 expression under this condition (Fig. 5c). H24R3 insult increased GAS5 and TSP-1 mRNA levels (Fig. 5c) and miR-21 mimics could suppress GAS5 and TSP-1 mRNA expression in H24R3-treated HK-2 cells, as well as TSP-1 protein abundance (Fig. 5d, f). These data revealed the negative regulation of GAS5 and TSP-1 by miR-21 could contribute to its anti-apoptotic effects in H/R-treated HK-2 cells. Interestingly, TSP-1 protein expression was downregulated in the setting of H6R0.5 and H24R3 (Figs. 4c and 5e), while its mRNA abundance was almost unaltered by H6R0.5 and upregulated by H24R3 insult (Fig. 5a, c).

We already clarified the role of GAS5 in cell apoptosis induced by H/R insult (H24R3) in our previous work^11^. The RT-PCR results showed that GAS5 siRNAs resulted in miR-21 upregulation accompanied by TSP-1 downregulation in H/R-treated HK-2 cells (Fig. 5g). Conversely, overexpression of GAS5 decreased the miR-21 abundance and increased TSP-1 expression remarkably (Fig. 5h). The regulation of TSP-1 protein levels by GAS5 and its effects on apoptosis in HK-2 cells have been fully confirmed in our previous study^11^. Hence, the regulation of miR-21 and TSP-1 by GAS5 could give rise to its pro-apoptotic effects in HK-2 cells.

### GAS5 acted as a ceRNA for miR-21 to target TSP-1

**Fig. 6:**
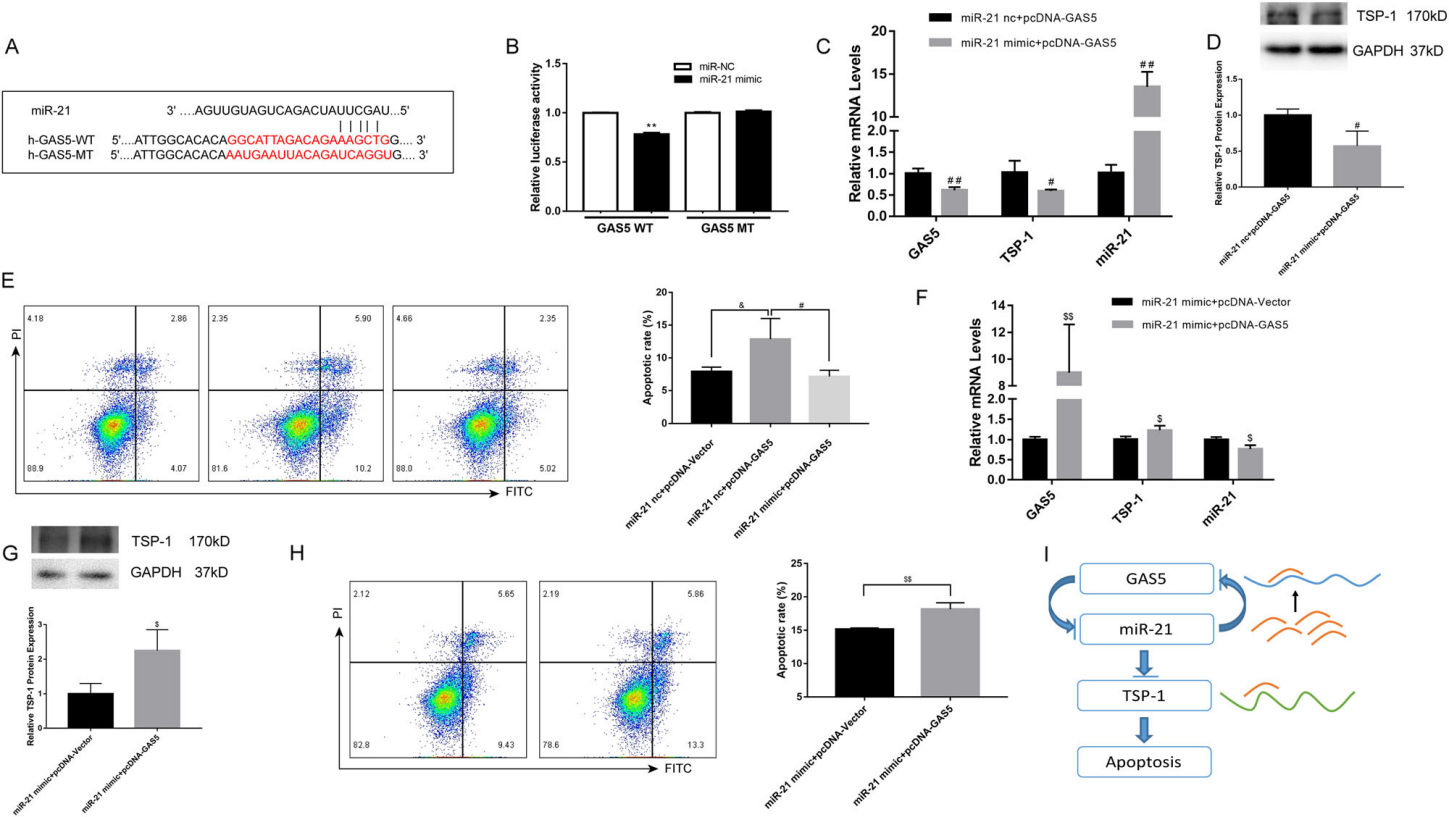
GAS5 served as a ceRNA of miR-21. **a** WT and MT GAS5 binding sites with miR-21 were present. **b** A dual luciferase reporter assay was performed to detect the predicted binding sites between GAS5 and miR-21. **c**, **d** Co-transfection of pcDNA-GAS5 with miR-21 mimic suppressed GAS5 levels, and inhibition of TSP-1 by miR-21 still existed under the condition of GAS5 overexpression. **f**, **g** Co-transfection of miR-21 mimic with pcDNA-GAS5 counteracted the inhibition of TSP-1 by miR-21 compared with co-transfection with pcDNA-Vector. **e**, **h** Cell apoptosis in HK-2 cells of co-transfections. **i** Schematic diagram of the hypothesis of this study. *N* = 3–4, ^**^P < 0.01 vs. miR-NC, ^#^*P* < 0.05, ^##^*P* < 0.01 vs. miR-21 nc+pcDNA-GAS5, ^$^*P* < 0.05, ^$$^*P* < 0.01 vs. miR-21 mimic+pcDNA-Vector, ^&^*P* < 0.05 vs. miR-21 nc+pcDNA-Vector.

Zhang’s study revealed that lncRNA GAS5 might contain a target site of miR-21^12^. The dual-luciferase reporter assay we performed showed that miR-21 mimic, but not miR-21 nc, apparently downregulated the luciferase activity of GAS5-WT but did not alter the luciferase activity of GAS5-MT (Fig. 6b). Our data above suggested that GAS5 might serve as a sponge for miR-21. Furthermore, to identify the potential mechanism of GAS5 that leads to H/R-induced apoptosis, we first performed co-transfection of pcDNA-GAS5 and miR-21 mimic or nc in HK-2 cells. As shown in Fig. 6c, d, the negative regulation of TSP-1 mRNA and protein expressions by miR-21 still existed under conditions of GAS5 overexpression. Co-transfection of pcDNA-GAS5 with the miR-21 mimic could markedly decrease GAS5 abundance and cell apoptosis compared with co-transfection with miR-21 nc (Fig. 6c, e). Next, co-transfection of miR-21 mimic and pcDNA-GAS5 or pcDNA-Vector showed that inhibition of miR-21 on TSP-1 expression could be rescued and cell apoptosis was aggravated by GAS5 overexpression (Fig. 6f–h). Therefore, GAS5, as a ceRNA of miR-21, rescued the silencing effect on its target, TSP-1, and promoted apoptosis during renal I/R injury (Fig. 6i).

## Discussion

LncRNA GAS5, a key regulator of cellular apoptosis, migration, and proliferation, has been widely studied in various types of tumors^13^. The present study indicated the relationship among GAS5, miR-21, and TSP-1 in the regulation of cell apoptosis under renal I/R condition and the underlying mechanisms.

Apoptosis is involved in the pathophysiology of ischemic AKI by complex gene regulations^1^. LncRNAs have been documented to have important apoptosis-related functions in tumor studies, including GAS5 as a tumor suppressor^14^. GAS5 interacts with the DNA-binding domain of the glucocorticoid receptor to suppress a number of anti-apoptotic genes, such as cIAP2^14^. After Lin et al. gave a landscape of lncRNAs induced by hypoxia, an increasing number of studies have explored the relationship between lncRNAs and AKI^15^. GAS5 could be heavily induced in renal tissues by I/R insult due to its pro-apoptotic function^11^. Downregulated GAS5 might be involved in the protective effects of delayed IPC.

It was recently discovered that lncRNAs functioned as miRNA sponges. Tian’s study indicated that lncRNA LINC00520 acted as a ceRNA to competitively inhibit miR-27b-3p during I/R injury^16^. Negative regulation between miR-21 and GAS5 was first found in breast tumors^12^. In our study, we found negative regulation between GAS5 and miR-21 in vitro and in vivo. Moreover, luciferase reporter assays showed that GAS5 acted as a sponge to bind directly to miR-21 with a putative miRNA response element. In addition to miR-21, it has been documented that GAS5 could bind to other miRNAs as a ceRNA, including miR-23a in gastric cancer^17^, miR-222–3p in papillary thyroid carcinoma^18^, and miR-137 in ischemic stroke^19^.

As another type of noncoding RNA, miRNAs have been associated with the etiology and pathogenesis of AKI. Godwin et al. first compared the genome-wide miRNA expression profile between I/R- and Sham-treated kidneys^20^. Several specific miRNAs, such as miR-24, miR-30a, miR-146, and miR-210, were also investigated in subsequent studies^21^. Accumulating evidence has elucidated that miR-21 protected renal epithelial cells against hypoxic and inflammatory injury and participated in renal protection of delayed IPC^2^, xenon preconditioning^22^, and delayed remote IPC^23^ in AKI. miR-21 in urine and plasma was associated with severe AKI after cardiac surgery^24^. miR-21 protected kidneys mainly by targeting pro-apoptotic genes such as PDCD4, PTEN, and FasL^25^. We found and confirmed a novel target of miR-21 that regulated apoptosis during AKI in our study.

TSP-1, an important matricellular glycoprotein, has multiple functions, including anti-angiogenesis, pro-fibrosis, pro-inflammatory, and pro-apoptosis in various cell types. TSP-1 in kidney tissues could be upregulated by ischemia^5^, unilateral ureteral obstruction^26^, and high glucose^27^, but it is rarely expressed in healthy kidneys. For the first time, Thakar et al. suggested that TSP-1 expression in renal proximal tubules was a novel regulator of I/R damage due to its pro-apoptotic function in ischemic AKI^5^. Additionally, the absence or disruption of TSP1-CD47 signaling protected mice from renal dysfunction and tubular damage^28^. Furthermore, TSP-1 could inhibit renal tubular epithelial cell proliferation and self-renewal after I/R injury via activation of its receptor CD47^29^. On the basis of our previous findings^4^, we further identified TSP-1 as a target gene of miR-21 in renal tubular epithelial cells in this study. The trend of TSP-1 expression after reperfusion in the current study was similar to the results of other studies, which was also consistent with the GAS5 expression levels. Our previous study suggested that the role of GAS5 in AKI is possibly related to TSP-1^11^, and we further confirmed that TSP-1 was positively regulated by GAS5 to promote apoptosis induced by renal I/R. Crucially, TSP-1 upregulation by GAS5 could be counteracted by miR-21 mimic, while the inhibition of TSP-1 expression by miR-21 could be rescued by GAS5 overexpression.

Summarily, we found that GAS5 could indirectly upregulate TSP-1 expression to promote apoptosis by interfering with the function of miR-21 as a ceRNA. Our results revealed novel information regarding the pathogenesis of ischemic AKI.

## Materials and methods

### Mouse model of renal ischemia/reperfusion injury and delayed renal ischemic preconditioning

Study protocols were approved by Institutional Animal Care Use Committee of Fudan University. All mice were divided into groups randomly. Briefly, 6- to 8-week-old male C57BL/6 mice were anesthetized with intraperitoneal sodium pentobarbital (80 mg/kg). Bilateral renal pedicles were clamped for 35 min in I/R group, followed by various reperfusion intervals. Sham-operated mice underwent anesthesia, laparotomy, and renal pedicle dissection only. Ischemic preconditioning (IPC) and I/R was performed by clamping the bilateral renal pedicles for 15 min and then for 35 min 4 days later, followed by 24 h reperfusion. LNA-modified anti-scramble or anti-miR-21 oligonucleotides (Exiqon, USA) (10 mg/kg) were administered intraperitoneally less than 1 h prior to IPC^2^.

miR-21 Tg+/0 mice were used at 8–10 weeks of age. miR-21 Tg0/0 mice from the same litter were used as controls in the studies. miR-21 Tg+/0 mice and Tg0/0 mice were subjected to renal ischemia for 35 min, followed by reperfusion for 24 h.

Kidneys and blood samples were collected at the indicated time. Serum creatinine were measured using a Quantichrom Creatinine Assay Kit (BioAssay Systems, USA).

### Histological analysis of renal injury

Kidney tissues were fixed in 4% paraformaldehyde, embedded in paraffin, cut into 4-μM-thick sections and stained with hematoxylin-eosin. Histopathological scoring was performed as previously described^11^.

### TUNEL staining

Paraffin-embedded kidney tissue sections were stained with an In Situ Cell Death Detection Kit (Roche, Switzerland). TUNEL-positive cells were counted at 200× magnification in 10 fields selected randomly from each slide.

In addition, a TUNEL FITC Apoptosis Detection Kit (Vazyme, China) was used to analyze apoptosis in mice kidneys subjected to I/R. Fluorescence microscopy was employed to detect apoptotic cells stained with green fluorescent dye.

### In situ hybridization of miR-21 and TSP-1

In situ hybridization (ISH) was carried out to reveal miR-21 and TSP-1 exposure in Sham- and I/R-induced kidneys. The procedure has been described previously^11^, and the primers are listed in Table 1.

**Table 1:** Primer sequences for RT-PCR, siRNA sequences for GAS5 and primer sequences for ISH probes.

mouse GAS5 forward	GGATAACAGAGCGAGCGCAAT
mouse GAS5 reverse	CCAGCCAAATGAACAAGCATG
human GAS5 forward	CTTGCCTGGACCAGCTTAAT
human GAS5 reverse	CAAGCCGACTCTCCATACCT
mouse TSP-1 forward	GACTCGGGACCCATCTATGA
mouse TSP-1 reverse	GGTTATGATTGGCAGCTGATG
human TSP-1 forward	GGCAAGGACTGCGTTGGT
human TSP-1 reverse	CACTTCACGCCGGCAAAG
18S forward	CGGCTACCACATCCAAGGAA
18S reverse	CCTGTATTGTTATTTTTCGTCACTACCT
human-si-GAS5 sense	CUUGCCUGGACCAGCUUAAUU
human-si-GAS5 antisense	UUAAGCUGGUCCAGGCAAGUU
miR-21 ISH probe forward	CGCGGGAATTCGATTtgtaccaccttgtcgggtag
miR-21 ISH probe reverse	GAATTCACTAGTGATgataccaaaatgtcagacagc
TSP-1 ISH probe forward	CGCGGGAATTCGATTaaagcctgcaagaaagacgc
TSP-1 ISH probe reverse	GAATTCACTAGTGATtgtttgttggccatggcatg

### Cell culture, hypoxia/reoxygenation treatment and transfection

Human renal proximal tubular epithelial (HK-2) cells were obtained from the American Type Culture Collection (ATCC, Manassas, VA) and cultured in Dulbecco’s modified Eagle’s medium/F12 supplemented with 10% fetal bovine serum. Cells were grown at 37 °C with 5% CO_2_ and passaged every 3–4 days.

Confluent HK-2 cells were exposed to hypoxia (1% O_2_, 5% CO_2_, and 94% N_2_) for 24 h, followed by 3 h of reoxygenation (H24R3), or to hypoxia for 6 h, followed by 0.5 h of reoxygenation (H6R0.5), according to different miR-21 expression levels.

GAS5 and negative control (NC) small interfering RNAs (siRNAs) or miR-21 nc and mimic were transfected into HK-2 cells followed by H24R3 treatment. HK-2 cells transfected with LNA-modified anti-miR-21 and anti-scramble (Exiqon, USA) were exposed to H6R0.5 insult. We overexpressed GAS5 by plasmid transfection. All transfections were performed using Lipofectamine 3000 (Invitrogen, USA) according to the manufacturer’s protocol. In view of cytotoxicity and adverse effects on apoptosis, the dosages of Lipofectamine 3000, plasmids and mimic(nc) were halved when assessing apoptotic rates of co-transfections.

### RNA extraction and real-time PCR

Total RNA was extracted from HK-2 cells and renal tissues using TRIzol reagent (Sigma). For GAS5 and TSP-1, RNA was reverse-transcribed to cDNA using PrimeScript RT Master Mix (Takara, Japan), followed by quantitative analysis using SYBR Premix Ex Taq (Takara, Japan) and an ABI 7500 real-time PCR system. 18S mRNA was used as an internal reference. For miR-21 analysis, RNA was reverse-transcribed into cDNA using miRNA-specific primers and a TaqMan MicroRNA Reverse Transcription Kit (Applied Biosystems, USA). Then, cDNA was amplified using TaqMan Universal PCR Master Mix. U6 was used as an endogenous control. Analysis was performed with the 2^−ΔΔCt^ method. The primers are listed in Table 1.

### Western blot analysis

Protein obtained using RIPA lysis buffer was separated by 8% polyacrylamide-SDS gels, transferred to PVDF membranes, blocked with 5% nonfat milk and then incubated with primary antibodies overnight at 4 °C followed by secondary antibodies, including horseradish peroxidase-conjugated anti-mouse immunoglobulin G (1:5000; Jackson ImmunoResearch Lab, USA). Primary antibodies included TSP-1 (1:100; Thermo Fisher, USA) and GAPDH (1:2000; Proteintech, USA).

### Luciferase reporter assays

A segment of the 3′UTR region of human TSP-1 mRNA, including the predicted miR-21 binding site, was amplified for cloning into the pGL3 basic plasmid downstream of the luciferase reporter gene. Luciferase activities were measured after the transfection.

A fragment from GAS5 containing the predicted miR-21 binding site was amplified and then cloned into a psi-CHECK2 luciferase vector (Promega, USA), which was named GAS5-wild-type (GAS5-WT). Its counterpart with a mutated miR-21 binding sequence was named GAS5-mutated-type (GAS5-MT). Then, GAS5-WT and GAS5-MT were co-transfected with miR-21 mimics or miR-21 nc. After transfection for 48 h, the relative luciferase activities were assessed by a Dual-Luciferase Reporter Assay System (Promega, USA).

### Flow cytometry

Apoptosis in HK-2 cells was evaluated by Annexin V-FITC/PI double staining (Invitrogen, USA). Cells were harvested and washed with 1× binding buffer and then incubated with Annexin V-FITC for 15 min, followed by PI staining for 5 min in the dark. Cell apoptosis was analyzed by flow cytometry (Invitrogen, USA).

### Statistics

Statistical analysis was performed using SPSS version 21.0. All data were expressed as mean ± SD. Comparisons of means between two groups were performed with unpaired Student’s two-sided t test. A *P* value of <0.05 was considered statistically significant.

## Supplementary information

authors contributions
