# Supplementary material for: LncRNA GAS5 promotes apoptosis as a competing endogenous RNA for miR-21 via thrombospondin 1 in ischemic AKI
Source: Cell Death Discov. 2020 Apr 2;6:19. doi: 10.1038/s41420-020-0253-8 (PMC7118150; doi:10.1038/s41420-020-0253-8)
Supplement: Supplementary file 1 — authors contributions [file 41420_2020_253_MOESM1_ESM.pdf]

DECLARATION OF CONTRIBUTIONS TO ARTICLE

ADMC

Manuscript Number:	Journal Name:	
CDDISCOVERY-19-0808	*Cell Death Discovery*	(the 'Journal')

Proposed Title of the Contribution:

LncRNA GAS5 promotes apoptosis as a competing endogenous RNA for miR-21 via thrombospondin 1 in ischemic AKI	(the 'Contribution')

Author(s):

Xuemei Geng, Nana Song, Shuan Zhao, Jiarui Xu, Yong Liu, Yi Fang, Mingyu Liang, Xialian Xu, Xiaoqiang Ding	(the 'Authors')

For all *CDDis*covery articles, each person named as an author in the published version must be able to show he or she has contributed substantially to the article.

Authorship credit should be based on 1) substantial contributions to conception and design, acquisition of data, or analysis and interpretation of data; 2) drafting the article or revising it critically for important intellectual content; and 3) final approval of the version to be published. Authors should meet conditions 1, 2 and 3.

Any person who cannot be shown to have made a substantial contribution to the article cannot be listed as an author in the final version. The name of any person who is deemed to have made a minor contribution can, however, appear in the Acknowledgments section of the article.

Please complete the table below to indicate the contributions of all named authors to the manuscript.

Author Full Name:	Specification of Contribution to the Manuscript:
Xiaoqiang Ding	conception and design, analysis and interpretation of data, revising it critically and final approval of the version to be published
Xialian Xu	design, acquisition of data, analysis and interpretation of data, drafting the article and revising it critically, final approval of the version to be published
Xuemei Geng	design, acquisition of data, analysis and interpretation of data, drafting the article and revising it critically, final approval of the version to be published
Nana Song	acquisition of data, analysis and interpretation of data, revising it critically, final approval of the version to be published
Shuan Zhao	design, acquisition of data, analysis and interpretation of data, revising it critically, final approval of the version to published
Jiarui Xu	acquisition of data, analysis and interpretation of data, revising it critically, final approval of the version to published
Yong Liu	conception and design, revising it critically, final approval of the version to published
Yi Fang	revising it critically, final approval of the version to published
Mingyu Liang	conception and design, revising it critically, final approval of the version to published

Please complete the table below to indicate the contributions of all named authors to the figures.

Figure 1:

Xuemei Geng did the animal model and examined the levels of serum Cre, GAS5, miR-21 and TSP-1. Shuan Zhao performed the ISH. Nana Song did the TUNEL assay.

Figure 2:

Xuemei Geng performed the IPC animal model and examined the levels of GAS5, miR-21 and TSP-1. Nana Song assessed the renal injury. Jiarui Xu did the TUNEL assay.

Figure 3:

Xuemei Geng performed the animal model and examined the levels of GAS5, miR-21 and TSP-1. Shuan Zhao assessed the renal injury. Xialian Xu did the TUNEL assay.

Figure 4:

Xuemei Geng generated the data and prepared the panel A-G. Xialian Xu did the relative animal experiments and prepared the panel H-M.

Figure 5:

Xuemei Geng performed the transfections and examined the levels of GAS5, miR-21 and TSP-1 with Xialian Xu. Jiarui Xu assembled the figure.

Figure 6:

Xuemei Geng and Xialian Xu completed the luciferase reporter assay. Xuemei Geng performed the transfections. Xuemei Geng and Jiarui Xu examined the levels of GAS5, miR-21 and TSP-1. Xialian Xu performed the flow cytometry.

Signed for and on behalf of the Author(s):	Print Name:	Date:
	Xiaoqiang Ding	11-07-2019
